# Dietary red palm oil supplementation reduces myocardial infarct size in an isolated perfused rat heart model

**DOI:** 10.1186/1476-511X-9-64

**Published:** 2010-06-18

**Authors:** Dirk J Bester, Krisztina Kupai, Tamas Csont, Gergu Szucs, Csaba Csonka, Adriaan J Esterhuyse, Peter Ferdinandy, Jacques Van Rooyen

**Affiliations:** 1 Department of Biomedical Sciences, Faculty of Health and Wellness Sciences, Cape Peninsula University of Technology, Bellville, South Africa; 2 Cardiovascular Research Group, University of Szeged, Szeged, Hungary; 3 Pharmahungary Group, Szeged, Hungary

## Abstract

**Background and Aims:**

Recent studies have shown that dietary red palm oil (RPO) supplementation improves functional recovery following ischaemia/reperfusion in isolated hearts. The main aim of this study was to investigate the effects of dietary RPO supplementation on myocardial infarct size after ischaemia/reperfusion injury. The effects of dietary RPO supplementation on matrix metalloproteinase-2 (MMP2) activation and PKB/Akt phosphorylation were also investigated.

**Materials and methods:**

Male Wistar rats were divided into three groups and fed a standard rat chow diet (SRC), a SRC supplemented with RPO, or a SRC supplemented with sunflower oil (SFO), for a five week period, respectively. After the feeding period, hearts were excised and perfused on a Langendorff perfusion apparatus. Hearts were subjected to thirty minutes of normothermic global ischaemia and two hours of reperfusion. Infarct size was determined by triphenyltetrazolium chloride staining. Coronary effluent was collected for the first ten minutes of reperfusion in order to measure MMP2 activity by gelatin zymography.

**Results:**

Dietary RPO-supplementation decreased myocardial infarct size significantly when compared to the SRC-group and the SFO-supplemented group (9.1 ± 1.0% *versus *30.2 ± 3.9% and 27.1 ± 2.4% respectively). Both dietary RPO- and SFO-supplementation were able to decrease MMP2 activity when compared to the SRC fed group. PKB/Akt phosphorylation (Thr 308) was found to be significantly higher in the dietary RPO supplemented group when compared to the SFO supplemented group at 10 minutes into reperfusion. There was, however, no significant changes observed in ERK phosphorylation.

**Conclusions:**

Dietary RPO-supplementation was found to be more effective than SFO-supplementation in reducing myocardial infarct size after ischaemia/reperfusion injury. Both dietary RPO and SFO were able to reduce MMP2 activity, which suggests that MMP2 activity does not play a major role in protection offered by RPO. PKB/Akt phosphorylation may, however, be involved in RPO mediated protection.

## Background

Little is known about the effects of dietary edible oil supplementation on myocardial infarct size. Previous studies demonstrated that dietary RPO supplementation offers protection against ischaemia/reperfusion injury by improved aortic output recovery [1-4]. Engelbrecht and co-workers (2006) found that 6 weeks of dietary RPO supplementation was associated with increased PKB/Akt and p38 phosphorylation and decreased phosphorylation of JNK, when compared to standard rat chow fed controls. This suggests that RPO may inhibit apoptosis through MAPK and PKB/Akt signaling pathways. RPO-supplementation was shown to increase cGMP levels early in ischaemia [3,4]. NO levels in myocytes of RPO supplemented rats was increased after hypoxic conditions when compared to standard rat chow fed controls. This suggests that RPO-supplementation upregulate NO-cGMP signaling. In subsequent studies by Van Rooyen and co-workers (2008) summarized these results in a review and proposed several possible protective pathways [5]. Engelbrecht and co-workers (2009) also found that the inhibition of the PI3K pathway by pharmaceutical drug, wortmanin (a specific PI3K inhibitor) led to a loss of the improved functional recovery after ischaemia in RPO supplemented groups [6]. However, the inhibition of PI3K by wortmanin could only reduce functional recovery in RPO supplemented groups partially, suggesting that there may be more than one pathway of RPO-mediated protection involved. These studies have shown that dietary RPO supplementation is able to increase reperfusion function. However, there is some evidence that contractile dysfunction in non-infarcted myocardial tissue may play a role in cardiac function early after ischaemia [7]. This necessitates the measurement of myocardial infarct size to determine the efficacy of dietary RPO-supplementation in offering protection against ischaemia/reperfusion injury.

Myocardial infarction is one of the leading causes of mortality globally [8-10]. Research has shown that a decrease in infarct size leads to improved ventricular function [11-13]. Furthermore, function was better in hearts with small infarcts a year after the myocardial incident, when compared to functional measurements at initial discharge from hospital [11]. There is however, uncertainty as to the reason why ischaemic myocytes are killed and this complicates the search for agents that may reduce myocardial infarct size.

Overproduction of reactive oxygen species (ROS) may be considered as one of the major causes of tissue death in myocardial infarction [14-18]. As RPO contains several antioxidant micronutrients, we hypothesize that the antioxidative action of RPO may indeed contribute to the protection offered by RPO against ischaemia/reperfusion injury.

Matrix metalloproteinases (MMPs) are calcium and zinc dependant, endopeptidases which facilitate cell migration and tissue remodelling [19]. Recently it was found that MMP2 plays a role in ischaemia/reperfusion injury to the heart [20]. Activation of MMP2 during an ischaemic insult is associated with poor recovery and larger infarct size of the heart [20-24]. However, inhibition of MMP2 may lead to improved myocardial recovery after ischaemia/reperfusion injury [25-29]. MMP2 may be activated by oxidative/nitrosative stress, which is associated with myocardial infarction [30]. Activation of MMP2 by ROS is achieved through redox modification of the regulatory region of the pro-MMP molecule [31,32]. This redox modification leads to the demonstration of activity in the 72 kDa isoform of MMP-2, which is normally a pro-MMP isoform. Activated MMP2 damages cardiomyocytes during reperfusion by cleaving the contractile protein regulatory element, troponin I or other structural proteins [33-38].

Our aims for this study were: 1) to measure infarct size in dietary RPO-supplemented rat hearts which have been exposed to ischaemia/reperfusion injury and 2) to determine the effects of dietary RPO-supplementation on myocardial MMP2 activation and PKB/Akt phosphorylation.

## Materials and methods

All rats received humane animal care in accordance with the Guide for the Care and Use of Laboratory Animals, published by the U.S. National Institutes of Health (NIH publication 8523, revised 1985).

### Experimental design

**Figure 1:**
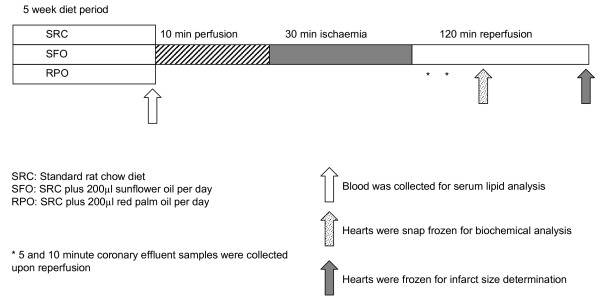
**Study design**.

Male Wistar rats were randomly divided into three groups and fed a standard rat chow diet (SRC), a SRC supplemented with RPO (200([0-9]+) μl/day), or a SRC supplemented with sunflower oil (SFO, 200([0-9]+) μl/day), for a five week period, respectively (Figure 1). The rats were individually housed to ensure that each animal received equal amounts of supplements, which were prepared on a daily basis in order to prevent spoiling. Supplements were added to rat chow that was ground finely with a pestle and mortar. All rats were allowed *ad libitum *access to food and water. For a brief description of supplements see Table 1.

**Table 1:** A brief breakdown of the content of oils, used as supplements in the diets of this study

	Sunflower oil	Red palm oil
**SFA (%)**	12	51
**MUFA (%)**	26	38
**PUFA (%)**	62	11
**Carotenoids (ppm)**	-	500
**Vitamin E (ppm)**	± 500	500-1100

After the feeding period, rat hearts were perfused for infarct size determination and analysis of Akt and ERK phosphorylation. Coronary effluent was collected during reperfusion for analysis of MMP2 and LDH activity. Serum obtained from the thoracic cavity of rats after removal of the heart was used for serum cholesterol and triglyceride determinations (Figure 1).

### Isolated heart perfusion

After the feeding period, rats were anaesthetized using diethyl ether. Hearts were rapidly excised, then mounted on a Langendorff perfusion apparatus and were perfused at 37°C at a constant pressure (75 mmHg) using a Krebs Henseleit buffer gassed with a mixture of 5% carbon dioxide and 95% oxygen. After mounting, hearts were subjected to 10 minutes of stabilization, followed by 30 minutes of normothermic global ischaemia and 120 minutes of reperfusion. At the end of the protocol ventricular tissue was frozen at -20°C overnight. Hearts for biochemical analysis were snap frozen after the 10 minute stabilization period and also after 10 minutes reperfusion.

### Infarct size determination

Frozen hearts were cut into 2 mm thick cross-sectional slices. These slices were stained in 2, 3, 5-triphenyltetrazolium chloride (TTC) for 10 minutes at 37°C. After TTC staining, the slices were transferred to a formalin solution for ten minutes and then placed in phosphate buffer (pH 7.4). Heart slices were then placed between two sheets of glass and scanned into a computer and analyzed using infarct size planimetry software (InfarctSize™ 1.0, Pharmahungary Szeged, Hungary). Infarct size was represented as percentage of the area at risk.

### LDH measurement

Lactate dehydrogenase (LDH) release from hearts was measured in coronary effluent samples that were collected for the first 5 min of reperfusion, using a LDH-P kit (Diagnosticum Zrt., Budapest, Hungary). The kit makes use of spectrophotometric measurement to determine the absorbance of substrate catalyzed by the enzyme.

### MMP2 zymography

Coronary effluent collected for the first 10 minutes of reperfusion was concentrated by ultra filtration using Amicon ultra filtration tubes. The concentrated coronary flow was then subjected to gelatin zymography. Gelatinolytic activities of MMPs were examined as previously described [39]. Briefly, polyacrylamide gels were copolymerized with gelatin, and a 10 μg protein was separated by electrophoresis in each lane. Following electrophoresis, gels were washed with 2.5% Triton X-100 and incubated for 20 hours at 37°C in incubation buffer. Gels were then stained with 0.05% Coomassie Brilliant Blue in a mixture of methanol/acetic acid/water and destained in aqueous 4% methanol/8% acetic acid. Zymograms were digitally scanned, and band intensities were quantified using Quantity One software (Bio-Rad, Hercules, CA).

### Western Blot

Heart tissue homogenized by adding homogenization buffer and PMSF to the sample. For phosphoprotein determination NaF and Na_3_VO_4 _were added to the sample after which samples were sonicated for 3 times 10 seconds and then centrifuged at 15000 rpm for 15 minutes. Protein concentration was determined by the bicinchoninic acid method.

Samples were diluted with Laemmli sample buffer and boiled, after which 40 μg of protein was separated by SDS-PAGE electrophoresis. After electrophoresis the proteins were transferred to PVDF membranes. Membranes where routinely stained with Ponceau in order to check for equal loading and sufficient transfer. Non specific binding was blocked by overnight incubation in 5% fat free milk in TBST. Membranes were then incubated with primary antibodies that recognize Erk p42/44 (Thr^202^/Thr^204^) and PKB (Thr^308^). Membranes were subsequently washed and incubated with secondary antibody. After thorough washing with TBST, membranes were covered with ECL and exposed to autoradiography films which were densitometrically analyzed.

### Serum Cholesterol and Triglyceride measurement

Serum chol and TG were measured using a test kit supplied by Diagnosticum Zrt. (Budapest, Hungary) as described previously [40].

### Statistical Methods

All values are presented as mean plus or minus standard error of the mean. Significance between groups was determined with one way ANOVA with Tukey Kramer post hoc test. *P *was considered significant if it was less than 0.05.

## Results

### Perfusion data and animal mass

The weight of rats from the RPO supplemented group was significantly less than that of the control groups after the feeding period (381 ± 7 g *versus *422 ± 8 g for the SRC and 403 ± 7 g for the SFO supplemented group). As animals were randomly divided into groups before the feeding period this is an indication that rats supplemented with RPO gained less weight in this time period (Table 2).

**Table 2:** Weight of rats, and rat hearts after a five week supplementation period.

	SRC	Sunflower oil	Red palm oil
**Animal mass (gram)**	422 ± 8	403 ± 7	381 ± 7 *#
**Heart mass (gram)**	1.57 ± 0.06	1.44 ± 0.04	1.40 ± 0.03 *

*P < 0.05 vs Ctrl, #P < 0.05 vs SFO

The weight of hearts isolated from the SRC group was significantly increased when compared to those of the RPO-supplemented group (1.57 ± 0.06g *versus *1.40 ± 0.03g). However, hearts from the SFO-supplemented group showed no difference when compared to the other two groups (1.44 ± 0.04g) (Table 2).

There were no significant differences between the groups with respect to heart rate and coronary flow before or after ischaemia. Coronary flow was significantly decreased after ischaemia in all groups when compared to pre-ischaemic values (Table 3).

**Table 3:** Functional parameters of hearts before and after ischaemia.

	Coronary effluent before ischaemia (ml/10 min)	Coronary effluent after ischaemia (ml/10 min)	Heart rate before ischaemia (BPM)	Heart rate after ischaemia (BPM)
**SRC**	151 ± 11	91 ± 4*	347 ± 16	158 ± 32
**SFO**	195 ± 20	127 ± 16*	349 ± 12	251 ± 40
**RPO**	152 ± 12	100 ± 6*	344 ± 11	328 ± 40

*P < 0.05 *versus *the same group before ischaemia

### Infarct size and lactate dehydrogenase (LDH)

**Figure 2:**
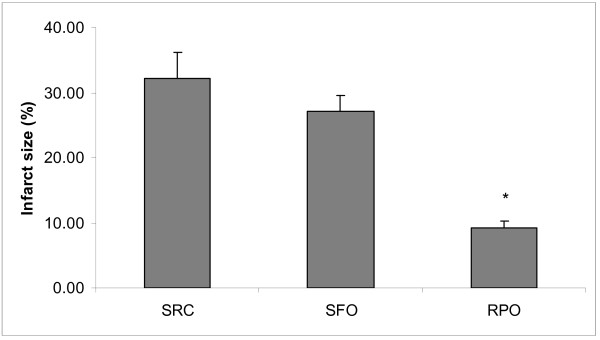
**Infarct size expressed as percentage of the area at risk of hearts that was subjected to 30 minutes global ischaemia and 120 minutes reperfusion**. *P < 0.05 vs SRC and SFO (n = 8)

Infarct size was significantly reduced in the RPO-supplemented group when compared to the SRC and the SFO-supplemented groups (9.1 ± 1.0 % *versus *30.2 ± 3.9 % for the SRC group and 27.1 ± 2.4 % for the SFO group, respectively). However, dietary SFO-supplementation did not reduce infarct size associated with ischaemia/reperfusion injury when compared to the SRC fed control rats (Figure 2).

**Figure 3:**
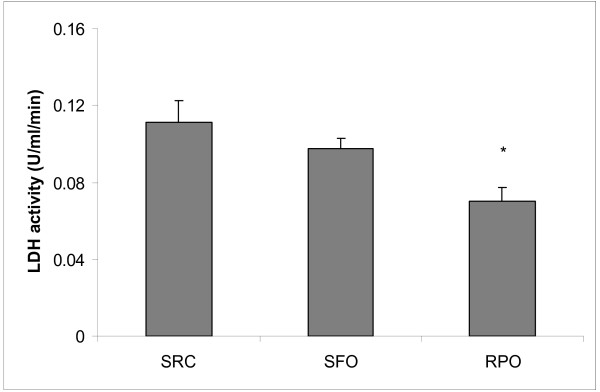
**Lactate dehydrogenase activity in coronary effluent collected from isolated perfused hearts during the first five minutes of reperfusion**. *P < 0.05 *versus *SRC (n = 8)

LDH levels in the reperfusion coronary effluent of the RPO supplemented group was significantly decreased when compared to the SRC group (0.11 ± 0.01 U/ml/min *versus *0.07 ± 0.01 U/ml/min) (Figure 3).

### Matrix Metalloproteinase 2 (MMP2)

**Figure 4:**
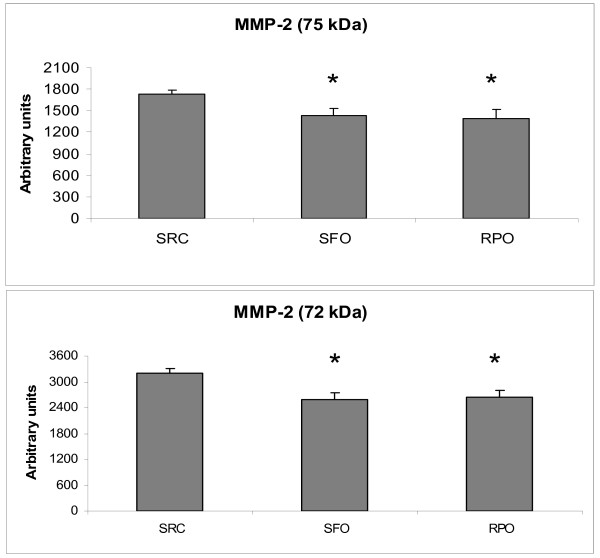
**MMP2 75 and 72 kDa isoform activities in coronary effluent of isolated perfused rat hearts, collected during the first ten minutes of reperfusion**. *P < 0.05 *versus *SRC (n = 8)

MMP2 activity in both the 75 kDa and 72 kDa isoforms were significantly decreased in the RPO and SFO-supplemented groups when compared to the SRC group (75 kDa: 1389 ± 124 arbitrary units and 1433 ± 103 arbitrary units *versus *1724 ± 69 arbitrary units; 72 kDa: 2635 ± 163 arbitrary units and 2597 ± 158 arbitrary units *versus *3201 ± 104 arbitrary units). There were no differences between the RPO-supplemented and SFO-supplemented groups for either the 75 kDa or the 72 kDa isoforms (Figure 4).

### Western blot analysis

**Figure 5:**
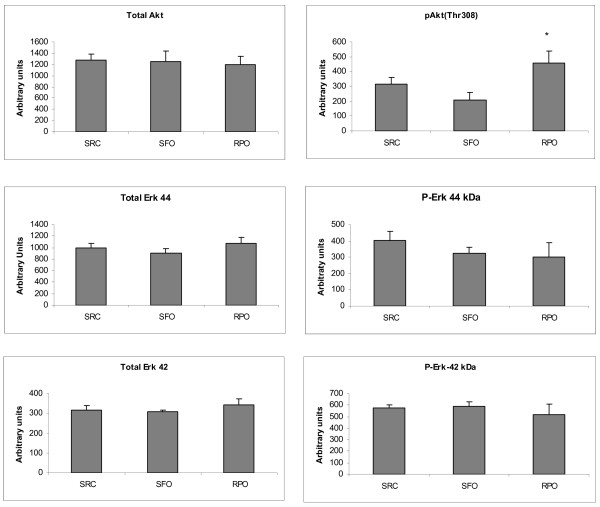
**Pro-survival kinase Western blots at 10 minutes into reperfusion**. *P < 0.05 vs SFO (n = 8)

The RPO supplemented group showed significantly increased Akt (thr) phosphorylation when compared to the SFO supplemented group (Figure 5). The total Akt for all groups were similar. There were no significant differences between any of the groups for total Erk 44 and 42 or phosphorylated Erk 44 and 42 (Figure 5).

### Serum lipid profile

**Figure 6:**
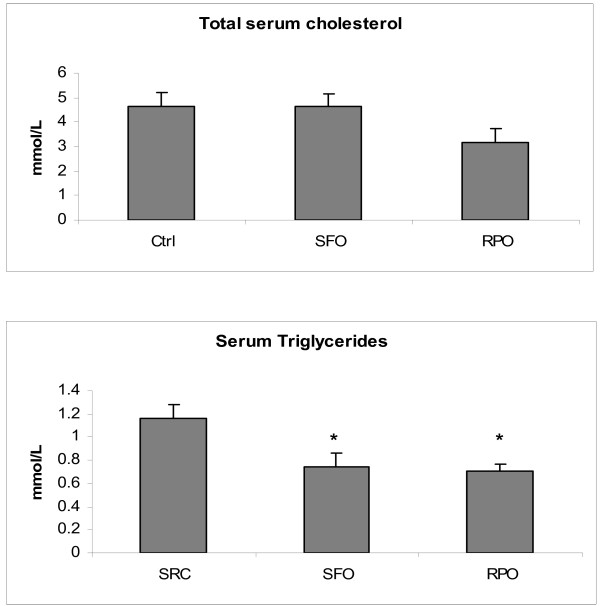
**Total serum cholesterol and triglyceride measurements performed after diet period**. *P < 0.05 vs SRC (n = 8)

The total serum cholesterol levels were similar in all the groups. There was, however, a significant decrease in serum triglyceride levels of both the SFO and RPO groups when compared to the SRC group (0.75 ± 0.12 mmol/L and 0.71 ± 0.06 mmol/L *versus *1.16 ± 0.12 mmol/L) (Figure 6).

## Discussion

Our results demonstrate that dietary RPO-supplementation was able to reduce myocardial infarct size. Sunflower oil supplementation however, was not able to reduce myocardial infarct size when supplemented to the diet in equal proportions as RPO, suggesting that the protection offered by RPO is not a result of additional energy added to the diet. This confirms results by Bester and co-workers (2006) which showed that RPO supplementation could offer protection against ischaemia/reperfusion injury in diets of different fat content when compared to isocaloric control groups. The reduction in infarct size in the RPO supplemented group also correlates well with functional results from previous studies where aortic output recovery was measured during reperfusion [1,3,4]. Infarct size however, may be used as a longer term predictor of myocardial recovery [11,13]. The results of this study together with the results of previous studies show for the first time that dietary RPO is able to offer protection against ischaemia/reperfusion injury by improving reperfusion function and reducing myocardial infarct size. Our coronary flow results show that neither dietary RPO supplementation, nor SFO supplementation was able to improve post ischaemic vascular action. These results are not in agreement with those of Esterhuyse and co-workers (2006) or Bester and co-workers (2006), where it was found that there was no singnificant differences between coronary flow before and after ischaemia in any group. The results of these studies should however, not be compared to the current study, as the model employed in the current study makes use of a harsher ischeamic temperature and time. Additionally the coronary flow was reported at 10 minutes reperfusion in the current study and at 25 minutes reperfusion in the above mentioned studies. The difference in heart weight between the RPO and SRC groups could be explained by the difference in body weight. These results indicate that dietary RPO-supplementation does not lead to increased weight gain or cardiac hypertrophy.

Reduction of MMP2 activity is normally associated with protection against ischaemia/reperfusion injury [21-24]. Under normal physiological conditions the 75 kDa and 72 kDa isoforms of MMP2 are inactive. These isoforms may however, display activity when partial cleavage of the autoinhibitory propeptide domain takes place in the presence of peroxynitrite and glutathione [31,32]. The fact that both RPO-supplementation and SFO-supplementation reduced the MMP2 activity suggests that both of these oils may offer some protection against ischaemia/reperfusion injury when supplemented to the diet. Alternatively, the reduction of MMP2 activity may not be the only pathway involved in RPO-mediated protection. The reduction of MMP2 (75 kDa and 72 kDa) activity suggests that oxidative stress has been reduced by the supplemented oils. However, dietary SFO-supplementation failed to decrease infarct size in this study. The reduction in MMP2 activity in the SFO-supplemented group was therefore not enough to offer protection against ischaemia/reperfusion injury. This suggests that MMP2 activity is not the only role player in the protection offered by RPO supplementation. Van Rooyen and co-workers (2008) proposed that several mechanisms synergistically offered RPO-mediated protection [5]. Our results suggest that reduction in MMP2 activity may together with other pathways such as 1) the NO-cGMP pathway, 2) the PKB/Akt pathway and 3) the inhibition of caspases, play a role in offering protection in dietary RPO supplemented hearts.

The reduced coronary effluent LDH levels in RPO supplemented rats confirmed that RPO supplementation was able to offer protection against ischaemia/reperfusion injury. This supports the infarct size result, as LDH release from the heart is a marker of myocardial tissue damage.

Our results also demonstrate that RPO is more effective than SFO in offering protection against myocardial ischaemia/reperfusion injury. This may be attributed to several possible mechanisms. One such mechanism may be the higher antioxidant content in RPO as compared to SFO [41,42]. The antioxidant content of RPO may offer an explanation for the improved protection in hearts of RPO-supplemented rats. Previous results have shown that dietary RPO-supplementation led to increased levels of cGMP production, accompanied by increased intracellular nitric oxide levels in myocytes [4]. These authors suggest that antioxidants present in RPO scavenged superoxide and thus lead to the conservation of nitric oxide. This would in turn lead to increased cGMP- and decreased peroxynitrite production [43-45]. This increase in cGMP and nitric oxide, accompanied by the decreased peroxynitrite is suggested to be one of the pathways of protection of dietary RPO-supplementation [1,4,5].

Serbinova and co-workers (1992) and Das and co-workers (2008) used the vitamin E fraction of palm oil, named tocotrienol rich fraction (TRF) as dietary supplement or introduced it into the perfusate of isolated perfused hearts [46,47]. These studies showed that TRF could offer protection against ischaemia/reperfusion injury. In the study by Das and co-workers (2008) the protective effects of TRF is ascribed to its ability to increase Akt phosphorylation. This places the Akt phosphorylation results of this study into perspective, as we were able to confirm that increased Akt phosphorylation takes place when RPO is supplemented to the diet when compared to a sunflower oil supplemented group. This is also in agreement with results by Engelbrecht and co-workers (2006) who showed that Akt is phosphorylated by dietary RPO-supplementation. In this study, Akt_ser473 _was shown to be phosphorylated, while the current study demonstrates that the Akt_thr308 _residue is also phosphorylated. Das *et al*. (2008) suggested that it is the tocotrienol content of palm oil that is responsible for Akt phosphorylation. This data supports our finding that RPO, which contains high levels of tocotrienols, could induce phosphorylation of Akt. SFO also contains vitamin E, but predominantly tocopherols [48]. We can therefore conclude from our studies that tocotrienols were responsible for Akt phosphorylation.

Furthermore, our results confirm previous studies which showed that RPO does not have hypercholesterolaemic effects [49,50]. RPO has also been shown to decrease triglycerides to a similar level as SFO. These results demonstrate that dietary RPO-supplementation does not have negative effects on the serum lipid profile normally associated with saturated fats.

## Conclusion

Our results demonstrate that dietary RPO-supplementation reduces myocardial infarct size when compared to SRC fed controls. We were also able to demonstrate that RPO supplementation is more effective in reducing myocardial infarct size than SFO fed group. MMP2 seems to have played at most, a minor role in the reduction of myocardial infarct size in this study, as its activity was reduced in both the RPO- and SFO-supplemented groups. The effects of reduced MMP2 activity may however contribute to RPO mediated protection against ischaemia/reperfusion by working synergistically with other mechanisms, as suggested by Van Rooyen and co-workers (2008). Our results confirm that Akt phosphorylation and possibly antioxidant activity of RPO may have been more effective in the protection offered by RPO against ischaemia/reperfusion injury.

## Competing interests

The authors declare that they have no competing interests.

## Authors' contributions

DB was involved in all experimental procedures for this manuscript and drafted the manuscript. KK played a major role in all the experimental procedures of this study. TC was involved with the design of the study and contributed towards the interpretation of the data and the revising of the manuscript. GS was involved with the experimental work performed towards this manuscript. CC was involved with the design of the study and contributed towards the interpretation of the data and the revising of the manuscript. AE was involved with the design of the study and contributed towards the interpretation of the data and the revising of the manuscript. PF was involved with the design of the study and contributed towards the interpretation of the data and the revising of the manuscript. JvR was involved with the design of the study and contributed towards the interpretation of the data and the revising of the manuscript. All the authors have read and approved the final manuscript.
